# Common bunt in organic wheat: unravelling infection characteristics relevant for resistance breeding

**DOI:** 10.3389/fpls.2023.1264458

**Published:** 2023-10-11

**Authors:** Magdalena Lunzer, Veronika Dumalasová, Kilian Pfatrisch, Hermann Buerstmayr, Heinrich Grausgruber

**Affiliations:** ^1^ Department of Agrobiotechnology, IFA-Tulln, University of Natural Resources and Life Sciences, Vienna, Tulln an der Donau, Austria; ^2^ Department of Genetics and Plant Breeding, Crop Research Institute, Prague, Czechia; ^3^ Department of Crop Sciences, University of Natural Resources and Life Sciences Vienna, Tulln an der Donau, Austria

**Keywords:** marker-assisted selection, organic farming, resistance breeding, *Tilletia tritici*, *Tilletia laevis*, *Triticum aestivum*

## Abstract

Common bunt caused by *Tilletia tritici* and *T. laevis* has re-emerged as a major threat to wheat yield and quality, especially in organic farming. Resistance against its causal agents is present in the wheat gene pool and provides the most economically efficient and sustainable way to combat the disease since seed treatments approved for organic farming are rare and do not always provide full protection. We tested a winter wheat diversity panel with 128 lines for common bunt resistance in Austria and Czechia, and evaluated the applicability of marker-assisted selection (MAS) via Kompetitive Allele-Specific PCR markers in genotypes with high variation in their genetic background. Field trials were conducted across two years and artificially inoculated with local bunt populations. The virulence patterns of these inocula differed between locations and only 15% of the tested genotypes showed stable resistance across test sites. Number and weight of bunt sori relative to the total number and weight of wheat grains in sampled ears revealed that partial infections of ears were frequently appearing. Forty-two breeding lines harboring combinations of four different resistance QTL were developed through MAS. Out of these, a quarter were resistant with a maximum of 5% common bunt incidence. On the other hand, only six out of 46 tested commercial cultivars and breeding lines showed no infection with common bunt, underlining the present scarcity of bunt-resistant cultivars for organic wheat production. By this study we showed that MAS is a useful tool to speed up the selection of resistant lines even in populations with highly diverse genetic backgrounds, and that it is efficient in pyramiding resistance loci and thereby improving the level of resistance.

## 1 Introduction

Common bunt of wheat caused by *Tilletia tritici* (Bjerk.) G. Winter (also called *Tilletia caries* (D.C.) Tul. & C. Tul.) and *T. laevis* J.G. Kühn (also called *T. foetida* (Wallr.) Liro) is experiencing a comeback on the fields after decades off the radar of researchers, breeders and farmers. The causal agents of this fungal disease belong to the division of the Basidiomycota and show differences in teliospore morphology. Despite this phenotypic variation, genetic studies suggest that *T. tritici* and *T. laevis* might be the same species (Carris, 2010; Sedaghatjoo et al., 2022). They also have identical life cycles with teliospores germinating at temperatures between 5°C and 20°C, relatively independent of light conditions (Lowther, 1950). This characteristic enables common bunt fungi to infect wheat seedlings also in the absence of continuous snow cover which is required for successful dwarf bunt (*T. controversa*) infections (Gassner and Niemann, 1954). Common bunt can therefore occur in both autumn- and spring-sown wheat given that temperatures after sowing are conducive for infection of the young seedlings (Goates and Bockelman, 2012). According to Hoffmann and Schmutterer (1983) optimum infection temperatures occur between 5°C and 10°C while Johnsson (1992) narrowed down the ideal temperature interval to 6-7°C. Especially the first ten days after sowing were the critical period for bunt infections in his field experiments conducted in Sweden. If environmental conditions were suitable during these first few days, bunt infections were high while temperatures, precipitation or snow cover after the initial ten days had no influence on infection levels (Johnsson, 1992). Hansen (1959) conducted experiments in controlled conditions in the greenhouse and found a lower sensitivity of common bunt spores to environmental temperatures. In her study, fungal hyphae were able to penetrate seedlings already four days after inoculation both at 3°C and 15°C. This highlights that the crucial period for bunt infections is restricted to a short time after seedling emergence. Even though the main inoculum source is usually contaminated grain, common bunt teliospores are also able to remain viable in the soil for years and thereby cause soil-borne infections of clean, healthy grain (Johnsson, 1990; Borgen, 2000; Goates and Bockelman, 2012). For this type of infection, the proximity between bunt spores and wheat seeds is essential. Only if teliospores are within a distance of 1 cm from sown grains, infection can occur (Johnsson, 1990). Borgen (2000) observed higher infection levels resulting from soil-borne teliospores two years after inoculum was brought into the soil compared to the first year. He concluded that this rise in infection levels was likely caused by teliospores being buried too deep in the soil by ploughing in the first year but being ploughed up again in the second year, resulting in closer proximity to the sown grains. Borgen (2000) concluded from his multi-year experiments that soil-borne common bunt spores can survive under the plough layer and remain viable enough to have practical implications under organic management for at least five years. Increased use of untreated seeds and minimum tillage practices are therefore boosting soil- and seed-borne diseases like common bunt if prevention measures such as appropriate hygiene in seed production, good crop rotation and cultivation of resistant varieties are neglected.

Resistance against bunt diseases is naturally occurring in the wheat gene pool but resistance genes are often found in landraces or non-adapted exotic genotypes. Based on phenotypic evaluation of reactions to different bunt races, a set of wheat differential lines harboring distinct types of resistances has been assembled by Hoffman and Metzger (1976) and extended by Goates (2012). Out of the 17 major resistance factors comprised in this differential set (*Bt1*-*Bt15*, *BtP* and *BtZ*), only four have been mapped to specific wheat chromosomes. The first molecular markers were devised for *Bt10* (Demeke et al., 1996; Laroche et al., 2000; Menzies et al., 2006) which has thereafter been widely used in breeding programs, especially in North America (Singh et al., 2016). Specifically, the D-genome seems to be important for bunt resistance in wheat since all major resistance factors hitherto mapped are located on group D chromosomes, i.e. *Bt9* (Steffan et al., 2017; Wang et al., 2019), *Bt10* (Menzies et al., 2006) and *Bt11* (Lunzer et al., 2023a) on 6D, and *Bt12* (Muellner et al., 2020) on 7D. For a long time, resistance to common bunt was seen as being only qualitative and based on gene-for-gene interaction (Hoffman and Metzger, 1976; Goates, 1996; Goates and Bockelman, 2012), but during recent years, also quantitative resistances have been identified. In fact, more quantitative trait loci (QTL) than major effect genes conferring bunt resistance are available to date. A special hotspot for bunt resistance QTL can be found on wheat chromosome 1B as many different mapping studies detected resistance conferring loci at different positions on 1B (Fofana et al., 2008; Wang et al., 2009; Dumalasová et al., 2012; Singh et al., 2016; Zou et al., 2017; Muellner et al., 2021; Iqbal et al., 2023; Lunzer et al., 2023a). It has been proposed that this chromosome harbors resistance genes *Bt4* and *Bt6* (Borgen et al., 2023a) but so far, these two factors have not been fine-mapped in dedicated populations. To make both resistance genes and QTL available for applied breeding, molecular markers for the selection of the respective chromosomal regions are essential. In earlier works, microsatellite markers (Fofana et al., 2008) or simple-sequence-repeats (SSR) (Dumalasová et al., 2012; Singh et al., 2016; Steffan et al., 2017) were used to construct linkage maps and determine molecular markers indicative of the respective chromosomal regions. Kompetitive allele-specific PCR-markers (KASP-markers) emerged more than a decade ago as a fast and easy alternative, suitable for screening large numbers of lines for the presence of resistance loci. Such KASP-markers have been developed and published for a range of bunt resistance sources (Wang et al., 2019; Muellner et al., 2020; Muellner et al., 2021). To enable efficient selection of QTL via marker-assisted selection (MAS), markers should ideally be flanking the chromosomal region mapped to harbor the respective locus. Finding such markers can sometimes be challenging if informative single nucleotide polymorphisms (SNPs) are unevenly distributed or generally scarce in certain chromosomal regions. Such situations have been described by Wang et al. (2019); Muellner et al. (2020) and Lunzer et al. (2023a). Despite these challenges, KASP markers which are not diagnostic but just located close to mapped resistance factors allow for MAS of the favorable loci. As chromosomal positions and markers for selection of more and more resistance loci become available, bunt resistance is being re-considered as a breeding goal in several wheat breeding programs, especially in those focused on organic farming.

Common bunt causes not only losses in grain yield through the replacement of grains by so-called ‘bunt balls’ (i.e. sori filled with fungal teliospores), but also deteriorates end-use quality by the typical rotten fish-like odor caused by trimethylamine, a volatile compound present in the teliospores (Hanna et al., 1932). Already low infection levels – Canadian studies mention 0.1% by volume and/or 0.05% by weight (Laroche et al., 2000; Menzies et al., 2006) – allow olfactory assessments as a means for common bunt detection (Börjesson and Johnsson, 1998). Another aspect of the typical bunt balls that has been discussed in a few works published in the mid-20^th^ century are partial infections of wheat kernels (Sampson, 1927; Gieseke, 1929; Gassner, 1938; Hansen, 1959). Information about this phenomenon is, according to our literature study, not found in any more recent publications on bunt diseases. Gassner (1938) questioned the until then widely accepted hypothesis that infections occurred through the ovules. Instead, he concluded from extensive microscopic analysis of partially bunted kernels that the ovules remained intact in partially infected grains but that they were seriously inhibited in their development and only ultimately replaced in cases of fully bunted kernels. Partially infected kernels were also investigated by Hansen (1959) who described that the pericarp was for the largest part replaced by bunt spores while endosperm and embryo were free from fungal cells. While Gassner (1938) considered fully bunted kernels the final stage of a transition from partial to full infections, Hansen (1959) assumes that the difference between fully and partially infected kernels is that only in the latter, successful pollination had occurred, leading to the development of embryo, endosperm and seed coat. Such partially infected kernels, mixed with completely healthy ones in a single ear, are hard to detect in a wheat field whereas fully bunted ears can be spotted with a little experience and training because of their modified appearance: They are usually shorter and spikelets are spread apart so that ears appear both flattened and stilted. If only partial infections occur, these symptoms are a lot harder to recognize or ears might even look completely healthy from the outside. Field trials with partially infected grains proved that the patches of bunt teliospores present inside otherwise healthy-looking kernels with unspoiled embryos were able to infect the seedlings emerging from these seeds. On the other hand, the removal of partially bunted grains from the seed lot via mechanical separation or washing was not possible (Gassner, 1938). These investigations were already conducted decades ago, but their conclusions can still be taken as valid today.

In order to add to the rather scarce knowledge about partial bunt infections, we wanted to study (i) whether partial infections occur in a diversity panel composed of multi-parent breeding lines and European cultivars, (ii) how measures for phenotypic evaluation of partial bunt infections were correlated to standard qualitative scoring of common bunt incidence, (iii) how common bunt infections in our panel differed between test locations in two European countries using different inocula, and (iv) whether marker-assisted selection can be applied as a tool for screening multi-parent breeding lines for bunt resistance QTL.

## 2 Materials and methods

### 2.1 Plant material

A panel of 128 wheat (*Triticum aestivum*) genotypes was assessed for different aspects of common bunt infection. A full list of all genotypes is available in **Supplementary Table S1**. The panel comprised 67 multi-parent winter wheat breeding lines developed at the Institute of Plant Breeding, BOKU, Tulln. The bunt resistance sources for these breeding lines were, on the one hand, three donors with mapped resistance loci, i.e. the differential line for bunt resistance gene *Bt12*, PI 199333 (Muellner et al., 2020) and the two cultivars ‘Blizzard’ and ‘Bonneville’ (Muellner et al., 2021). On the other hand, registered cultivars with unmapped bunt resistances were used. The donor lines (i.e. S5.58 derived from the cross Blizzard/Rainer, P101.30 derived from Bonneville/Rainer, and P106.24 derived from PI 11933/Rainer) were crossed to cultivars registered in various European countries provided by partners from the ECOBREED project. Depending on the number of crosses, each breeding line comprised between two and ten different genotypes in its pedigree. In addition, a set of 46 registered cultivars and commercial breeding lines originating from different countries was included in the test panel to evaluate the presence of bunt resistance in breeding programs across Europe. For monitoring the virulence of the applied bunt inocula across years, we also included the bunt differential set consisting of 14 wheat accessions each indicative for one of the known bunt resistance types (*Bt1* to *Bt13*, plus *BtP*) (Goates, 2012). Genotypes for *Bt14* and *Bt15* were excluded as these are tetraploid durum (*T. durum*) wheats. Instead, we included the susceptible controls ‘Heines VII’ (*Bt0*) and ‘Capo’.

### 2.2 Field trials

Artificially inoculated field trials were conducted in two locations in Austria and the Czech Republic. The experimental site in Austria was located in Tulln (48°19’05’’N, 16°04’10’’E) at an elevation of 177 m a.s.l. Mean annual temperature and precipitation in 2021 and 2022 were 10.2°C and 11.2°C, and 450 mm and 504 mm, respectively. Seed samples were artificially inoculated before sowing using a suspension of common bunt teliospores in a solution of 2% methylcellulose in water following a protocol adapted from Goates (1996) and Muellner et al. (2020). Teliospores were gained from infected wheat ears harvested in field trials of the previous seasons, cleaned from all plant residues and stored in a dry place at room temperature. When harvesting the infected ears, a wide range of medium infected genotypes (20-50% infection) was used as spore sources to avoid unintended selection and to ensure that the inoculum represented the local bunt population. The spore suspension for artificial inoculation was applied in a concentration of 0.09 g of spores (= 0.3 mL of spore suspension) per 10 g of seeds and distributed onto the seeds by shaking. Double-rows of 1.6 m length and spaced 25 cm apart were sown in the first two weeks of November. In 2021 and 2022, 98 and 84 genotypes were tested in Tulln, respectively. Herbicide treatment and fertilizer applications were carried out following standard agricultural practices. The experiments were laid out as augmented designs with two replicates for check cultivars and unreplicated test entries in both years.

Experimental fields in the Czech Republic were located at the Crop Research Institute in Prague-Ruzyne (50°05’05’’N, 14°17’58’’E) at 280 m a.s.l. Mean annual temperatures in 2021 and 2022 were 9.1°C and 10.1°C, respectively. Annual precipitation was 835 mm and 867 mm in the two test years, respectively. Seed samples were inoculated by shaking 250 seeds of each genotype together with 0.1 g of common bunt teliospores in an Erlenmeyer flask by hand for one to two minutes. Teliospores originated from a mixture of two Czech common bunt samples that were collected in 2014 and re-inoculated since then on the susceptible variety ‘Heines VII’. Field plots were sown by hand in mid-October as double-rows of 1 m length and spaced 20 cm apart. In 2021, 55 genotypes were tested and in 2022, 60 genotypes were tested. Weed removal in the field experiments was done by hand; no fertilizer or pesticides were applied. The trials were laid out as unreplicated randomized designs in both years.

### 2.3 Disease scorings

Common bunt infections (CB) were scored as disease incidence in 150 randomly selected ears per plot (Austria) or all ears per plot (Czech Republic) and the results were converted to percentages. The different number of scored ears between the two locations was due to the smaller plot size in Czechia, resulting in less than 150 ears for some plots. Ears were cut open and recorded as infected if a single bunt ball was spotted. If an ear was not obviously completely infected, a diagonal cut was first applied in the upper third of the ear and then a second diagonal cut was performed in the lower third of the ear to ensure that partial infections would be recognized. Scoring was done at the time of ripening between growth stages BBCH 80 and 89 in June and July.

In the Austrian field trials, 50 randomly chosen, non-cut ears were harvested from each plot after incidence scoring and subjected to further analyses. First, the number of bunt sori (BS) relative to the total number of ovules in the ear (i.e. healthy kernels plus bunt sori) was determined by manually removing all grains and bunt balls from wheat spikes. Bunt sori were then weighed and their weight relative to the total yield of the ear (i.e. healthy kernels plus bunt sori) was assessed (WBS). BS and WBS were determined on 82 of the 98 genotypes tested in 2021 and on 66 of the total 84 genotypes tested in 2022.

### 2.4 Marker-assisted selection in multi-parent breeding lines

MAS for known bunt resistance QTL was applied in the development of 42 out of the total 57 multi-parent breeding lines using KASP markers. Selection was carried out for four loci on chromosomes 1A, 1B, 7A and 7D which were mapped in the bunt resistant cultivars ‘Blizzard’ and ‘Bonneville’ (Muellner et al., 2021). ‘Blizzard’ was present in the pedigree of all 42 lines and 4 lines additionally contained ‘Bonneville’ as a parent (**Supplementary Table S1**). The 42 breeding lines originated from ten crosses which were conducted between nine pre-selected breeding lines, themselves originating from either three-way or four-way crosses, which harbored bunt resistance loci in heterozygous allelic states. The pre-selection of these nine lines based on their heterozygosity at the resistance loci was carried out using 14 KASP markers published by Muellner et al. (2021). Progeny from the ten crosses between the heterozygous breeding lines had complex pedigrees consisting of up to ten different genotypes. As this led to a loss of polymorphism for some of the KASP markers applied in the pre-selection, a slightly different set of markers with similar physical positions had to be used for MAS in the multi-parent breeding lines (**Supplementary Table S2**). We generally aimed to use markers at flanking positions of the QTL regions to achieve good selection accuracy. The full list of markers used for MAS in each genotype is available in **Supplementary Table S3**.

Prior to the screening with KASP markers, DNA was extracted from fresh leaf samples of eight to 14 plants per cross following a protocol adapted from Saghai-Maroof et al. (1984). DNA concentrations were normalized to 50 ng µL^-1^ and PCR reactions were carried out following the protocol for KASP PCR provided by LGC Biosearch Technologies (Berlin, Germany). Allelic discrimination results were obtained by reading fluorescence signals with a CFX384 TM Real-Time PCR Detection System (Bio-Rad Laboratories, Inc., Hercules, CA).

### 2.5 Data analysis

All statistical analyses were carried out in R (R Foundation for Statistical Computing, Vienna, Austria). Correlations between trials were calculated using Pearson’s correlation coefficient. Analysis of variance (ANOVA) was carried out for individual locations separately using a model of the form


$$P_{ijk}=\mu +G_{i}+E_{j}+GE_{ij}+e_{ijk}$$

where $P_{ij}$ is the phenotypic value observed for the respective trait, μ is the grand mean, $G_{ij}$ is the genotype effect of the $i^{th}$ line, $E_{j}$ is the effect of the $j^{th}$ environment (i.e. year), $GE_{ij}$ is the genotype-environment interaction of the $i^{th}$ genotype with environment *j* and $e_{ij}$ is the residual effect. For analysis across both locations, the model was extended to


$$P_{ijk}=\mu +G_{i}+E_{j}+L_{k}+GE_{ij}+GL_{ik}+EL_{jk}+e_{ijk}$$

where $L_{k}$ is the effect of location *k*, $GL_{ik}$ is the interaction effect between genotype *i* and the $k^{th}$ location and $EL_{jk}$ is the interaction between the $j^{th}$ environment and location *k*. All effects were modeled as random except for the grand mean, which was treated as a fixed effect. Models were fit using R package *breedR* (Muñoz and Sanchez, 2020) with the *remlf90* function. Broad-sense heritability (‘operative heritability’) was calculated following Strube (1967) as


$$H^{2}=\frac{\sigma_{G}^{2}}{\sigma_{G}^{2}+\frac{\sigma_{G\times E}^{2}}{n_{E}}+\frac{\sigma_{e}^{2}}{n_{E}}}$$

with $\sigma^{2}$ as the genotypic variance, $\sigma_{G\times E}^{2}$ as the genotype-environment interaction, $\sigma_{e}^{2}$ as the residual variance and $n_{E}$ as the number of test locations.

## 3 Results

### 3.1 Phenotypic evaluation of common bunt infections

#### 3.1.1 Differential set and cultivars

To monitor the virulence spectrum of the applied inoculum, the bunt differential set consisting of 14 differential lines plus two susceptible controls was tested in both locations (**Table 1**). Inocula used for artificial infection showed different virulence patterns between Austria and the Czech Republic. The Austrian inoculum was not virulent (0-1% CB) against *Bt1*, *Bt8*, *Bt11* and *Bt12* and showed low aggressiveness against *Bt4*, *Bt5*, *Bt6* and *Bt9* (1-10% CB) across two years (2021-2022). Infection levels were generally elevated in 2022 compared to 2021 in Austria. Qualitative differences were observed for *Bt5* and *BtP* with differential lines for these two genes being resistant in 2021 but infected in 2022. In the Czech Republic, the inoculum was avirulent to *Bt8*, *Bt9*, *Bt10*, *Bt12*, *Bt13* and *BtP* and showed low aggressiveness against *Bt11* in 2021. The bunt differential set was not tested in the Czech Republic in 2022 but also before in 2019 and 2020 (see **Supplementary Table S4** for all results).

**Table 1:** Common bunt incidence (%) for genotypes of the bunt differential set across two years and locations: Austria (Tulln, AT) in 2021 and 2022; Czech Republic (Prague, CZ) in 2021.

*Bt* gene	Name	Accession	AT 2021	AT 2022	CZ 2021
susceptible	Capo	01C0104425	70.0	91.5	–
*Bt0*	Heines VII	PI 209794	–	–	50.9
*Bt1*	Sel. 2092	PI 554101	0.0	0.0	39.9
*Bt2*	Sel. 1102	PI 554097	56.0	96.0	15.5
*Bt3*	Ridit	CItr 6703	10.3	18.0	11.9
*Bt4*	Turkey	PI 11610	1.3	0.0	60.6
*Bt5*	Hohenheimer	CItr 11458	0.7	10.0	63.0
*Bt6*	Rio	CItr 10061	1.7	1.0	21.8
*Bt7*	Sel 50077	PI 554100	35.3	98.0	48.2
*Bt8*	M72-1250	PI 554120	1.0	0.0	0.0
*Bt9*	R63-6968	PI 554099	9.0	6.0	0.0
*Bt10*	R63-6982	PI 554118	32.7	44.0	0.0
*Bt11*	M82-2123	PI 554119	0.0	0.0	8.2
*Bt12*	1696	PI 119333	0.0	0.0	0.0
*Bt13*	Thule III	PI 181463	19.7	22.0	0.0
*BtP*	7838	PI 173437	0.0	20.0	0.0

Two different susceptible controls were used in the two locations, i.e. ‘Capo’ in Tulln and ‘Heines VII’ in Prague. Accession codes refer to the USDA-ARS Germplasm Resources Information Network (GRIN) (see https://www.ars-grin.gov/) and GRIN Czech for cv. 'Capo' (https://grinczech.vurv.cz/gringlobal/).

**Figure 1:**
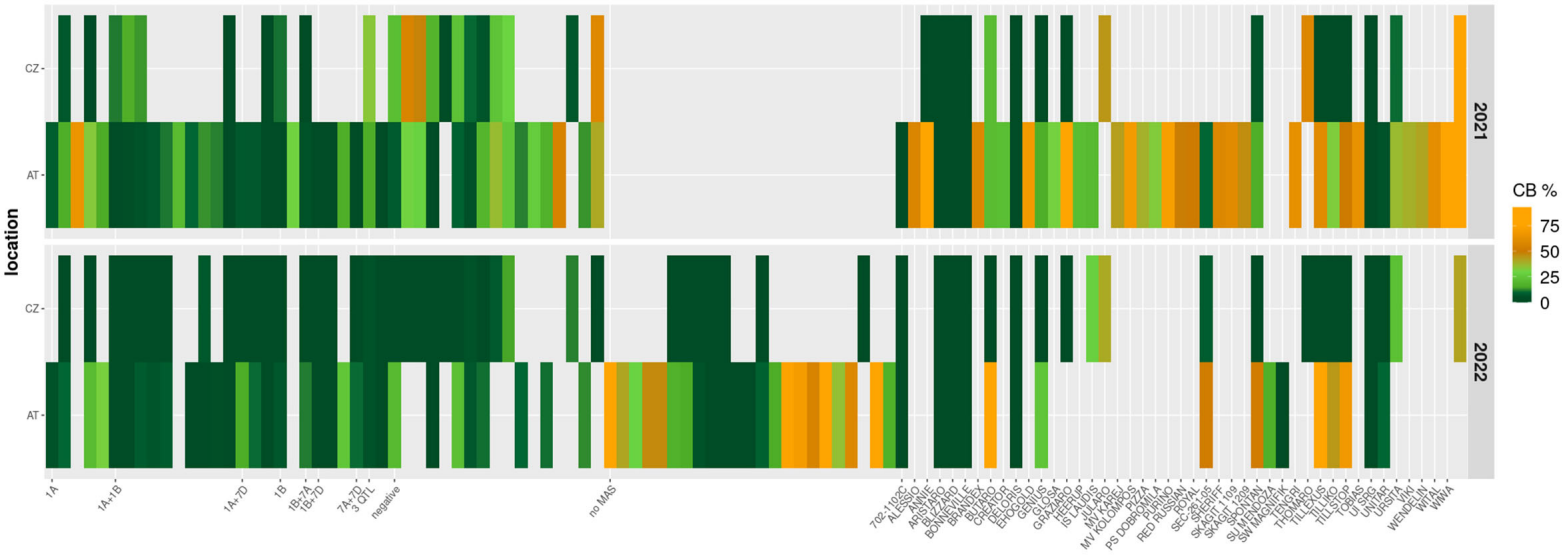
Heatmap of common bunt incidence (CB, %) across two years (2021, row 1 and 2022, row 2) and two locations (AT: Tulln, Austria; CZ: Prague, Czech Republic) for a diversity panel of 113 wheat genotypes. The left-hand side of the heatmap shows lines harboring different QTL according to marker-assisted selection using KASP markers; the chromosomal locations of the QTL are indicated on the x-axis. These QTL are known bunt resistance loci mapped by Muellner et al. (2020); Muellner et al. (2021) and originate from cultivars ‘Blizzard’ and ‘Bonneville’. Lines which were negatively selected and included as negative controls are indicated as “negative”, lines which were not subjected to MAS are indicated by “no MAS”, and a panel of cultivars and breeding lines is shown on the right-hand side with genotype names indicated on the x-axis.

Out of the 46 commercial cultivars in the panel, five (i.e. ‘Aristaro’, ‘Blizzard’, ‘Bonneville’, ‘Deloris’, ‘UI SRG’) showed resistance to common bunt across years and/or locations with up to 5% infection and one genotype (i.e. ‘Unitar’) had up to 10% incidence. All other cultivars were moderately to highly infected (**Figure 1**; **Table S1**).

#### 3.1.2 Evaluation traits of common bunt infections

High variation was observed in the levels of CB in both test locations (**Table 2**), ranging between 0 and 98% in the Austrian field trials and between 0 and 91.5% in the Czech experiments. Based on scorings in 2021, multi-parent breeding lines and cultivars that showed elevated infection levels and therefore did not qualify as interesting material for resistance breeding were excluded from the panel to enable testing of additional breeding lines and cultivars in 2022 (**Figure 1**). This led to a lower mean CB in both locations in 2022 because many highly susceptible cultivars were eliminated from the trials in this year. This down-shifting of average infection levels also occurred in Austria although CB was generally elevated by approximately 50% in 2022 due to environmental conditions highly favorable for bunt infections as obvious from higher CB values in the bunt differential set (**Table 1**). High variation was observed between the difference of CB to BS scores of individual genotypes, ranging from –24.9% to 27.7% in 2021 and between -5.1% and 48.3% in 2022 (**Table 2**). The negative relationships between CB and BS in 2021 were primarily due to four cultivars (i.e. ‘Alessio’, ‘Sheriff’, ‘Tillexus’ and ‘Tillstop’) which had high levels of CB but the number of sori relative to the total number of grains was even higher. These four cultivars were excluded from the 2022 trials. On average, CB scores were 4.1% (2021) and 7.4% (2022) higher than BS scores and 14.6% (2021) and 12.8% (2022) higher than WBS scores. While BS was on average 10.3% higher than WBS in 2021, this ratio dropped to 5.4% in 2022.

**Table 2:** Minima, maxima and mean values of common bunt scorings in individual locations and years: common bunt incidence (CB) in 150 ears per plot (Austria) or all ears per plot (Czech Republic); number of bunt sori relative to the total number of grains in 50 ears per plot (BS); and weight of bunt sori relative to the total grain weight of 50 ears per plot (WBS).

	Austria			Czech Republic		
	Min	Max	Mean	Min	Max	Mean
2021						
CB	0	81.3	25.5	0	91.5	18.9
BS	0	88.6	24.2			
WBS	0	74.7	13.3			
CB to BS	-24.9	27.7	4.1			
CB to WBS	0	47.4	14.6			
BS to WBS	0	29.0	10.3			
2022						
CB	0	98	22.1	0	50.5	5.4
BS	0	91.1	13.5			
WBS	0	81.2	8.1			
CB to BS	-5.1	48.3	7.4			
CB to WBS	0	53.4	12.8			
BS to WBS	0	24.6	12.8			

All values given as percentages, as well as differences between scorings of each trait relative to the other two.

### 3.2 Heritabilities and trait correlations

**Figure 2:**
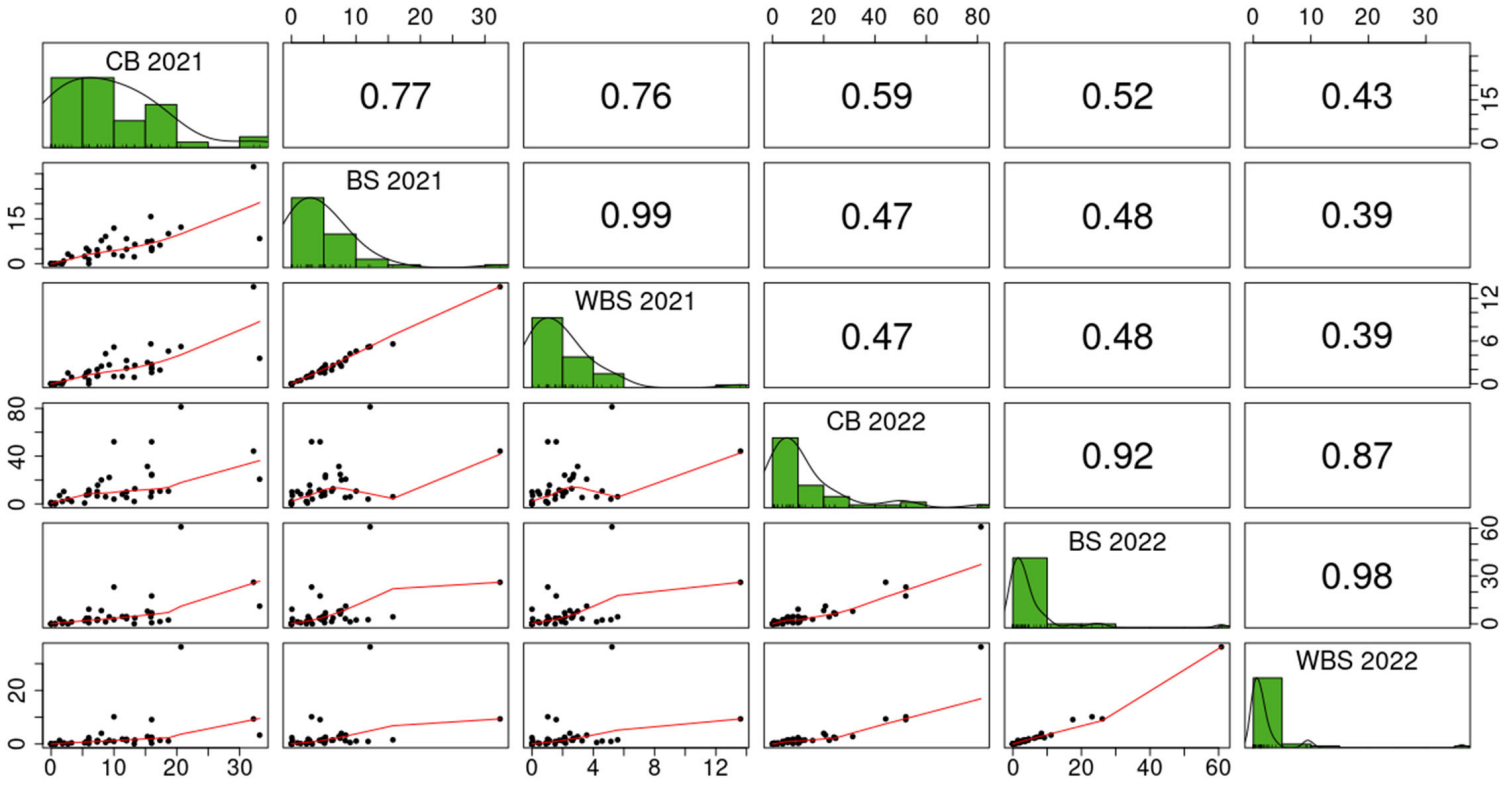
Scatterplots (below diagonal), histograms (diagonal) and Pearson’s correlation coefficients (above diagonal) for different common bunt infection traits evaluated in 2021 and 2022 in Austria: CB, common bunt incidence in 150 ears per plot; BS, number of bunt sori relative to the total number of kernels in 50 ears per plot; WBS, weight of bunt sori relative to the total grain weight in 50 ears per plot. All values in percentages.

ANOVA results showed that the largest part of the total phenotypic variation in CB as well as in BS was explained by the genotype if data were analyzed for each location separately (**Table 3**). For WBS, the residual component explained the largest part of the total phenotypic variance, followed by the genotype by environment interaction and the genotypic variance. When analysis was performed across trial sites, the largest part of the variation was also accounted for by the residual variance, followed by the interaction of year (environment) and location. Broad-sense heritability estimates were highest for CB (*H²* = 0.68 in Czech trials and *H²* = 0.63 in Austrian trials) and lower for BS (*H²* = 0.59) and WBS (*H²* = 0.44). Both ANOVA and estimation of broad-sense heritability were calculated on reduced data sets taking only genotypes into account that were tested in both years and/or locations, respectively. The same subsets of 42 (Austria), 40 (Czech Republic) and 22 (across years and locations) genotypes were used to estimate Pearson’s correlation coefficients between the different traits. Correlation coefficients for CB between 2021 and 2022 were similar between locations and significant at *α* = 0.001 (*r* = 0.59 in Austria and *r* = 0.63 in the Czech Republic; **Figure 2**). Correlation coefficients for BS and WBS were lower and significant at *α* = 0.01 (BS: *r* = 0.48; WBS: *r* = 0.39). In 2022, correlation coefficients between CB and BS/WBS were higher than in 2021. In addition, CB was more correlated to BS than to WBS in 2022, while correlation coefficients between CB and the two bunt sori parameters were almost equal in 2021. No significant correlation was observed for CB scorings between the two test sites in any year.

**Table 3:** Variance components and broad-sense (‘operative’) heritability estimates for common bunt assessment in individual locations and across locations.

Location	Trait ^a^	*σ_G_ ^b^*	*σ_E_*	*σ_L_*	*σ_GxE_*	*σ_GxL_*	*σ_ExL_*	*σ_error_*	*H²*
Austria	CB	81.2	5.6		39.6			55.7	0.63
	BS	29.9	0.02		17.3			24.5	0.59
	WBS	5.7	0.01		5.9			8.5	0.44
Czechia	CB	186.9	73.6		63.6			108.7	0.68
Across	CB	8.9	0.02	10.8	0.5	18.4	60.3	80.9	0.18

a Common bunt assessments: CB, common bunt incidence in 150 ears per plot (Austria) or all ears per plot (Czech Republic); BS, number of bunt sori relative to the total number of grains in 50 ears per plot; WBS, weight of bunt sori relative to the total grain weight of 50 ears per plot.

b variance components for: σ_G_, genotype; σ_E_, year (environment); σ_L_, location; σ_GxE_, genotype by environment interaction; σ_GxL_, genotype by location interaction; σ_ExL_, environment by location interaction; σ_error_, residual; *H*², broad-sense (‘operative’) heritability.

For within-location analyses, 42 and 40 genotypes tested in both years were included in the Austrian and Czech data, respectively. Analysis across locations comprised 22 genotypes that were tested in both years and locations.

### 3.3 Marker-assisted selection in multi-parent breeding lines

**Figure 3:**
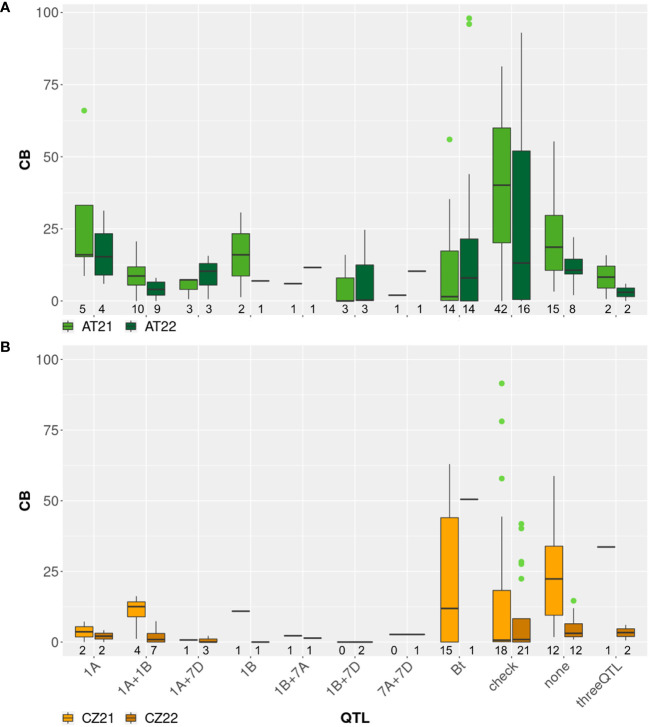
Boxplots showing common bunt incidence (CB, %) in different sub-groups of a diversity panel in field trials in **(A)** Austria and **(B)** Czech Republic in 2021 and 2022: genotypes identified with marker-assisted selection (MAS) to harbor different known bunt resistance loci originating from ‘Blizzard’ or ‘Bonneville’ (Muellner et al., 2020; Muellner et al., 2021) are indicated by the chromosomal locations of resistance QTL or the designation “threeQTL” on the x-axis; genotypes harboring no QTL according to MAS are marked as “none”; results for the bunt differential set including differentials for *Bt0*-*Bt13* and *BtP* are shown in group “Bt”; registered cultivars and breeding lines are shown as group “check”. The number of genotypes tested per group, location and year is indicated below the respective box; outliers are shown as dots.

For each cross, between eight and 14 progenies were screened with two to six KASP markers for one to four different bunt resistance QTL (**Supplementary Table S2**). If possible, two flanking markers for each QTL were used, but due to missing polymorphisms, some QTL could only be tested for with a single marker or could not be selected at all. Between two and four F_2_ lines from each cross were positively selected to harbor different QTL or combinations of QTL. A set of eight to 15 negatively selected lines were included in each test year as a control panel (**Figure 3**). In 2021, nine breeding lines out of 42 tested in Austria were found to be resistant with less than 5% CB and two of these were also completely resistant in the Czech Republic. In 2022, eleven lines out of 33 tested in Austria showed resistance and nine of these lines were also resistant in the Czech Republic. Six of these lines were tested in both seasons in Austria and one season in the Czech Republic and showed stable resistance across years and environments. These genotypes all harbored combinations of two or three different bunt resistance loci according to MAS results (**Supplementary Table S1**). In both test locations, genotypes selected to harbor bunt resistance loci were on average more resistant compared to negative controls and cultivars (**Figure 3**).

## 4 Discussion

A diversity panel consisting of the bunt differential set, a range of cultivars and breeding lines from European breeding companies and experimental multi-parent breeding lines developed at the Institute of Plant Breeding, BOKU, Tulln, was analyzed for common bunt resistance in two environments. The panel comprised a total of 128 genotypes out of which several subsets were used to assess different characteristics of common bunt infections. Specifically, for the multi-parent breeding lines, we aimed at determining differences between standard scoring and two alternative methods providing more detailed information about the degree of infection in individual ears.

### 4.1 Partially bunted ears

Scoring of common bunt incidence is usually done by cutting wheat ears and checking for the presence of bunt balls. Incidence is then scored in a qualitative manner, recording an ear as infected if at least a single bunted spikelet is spotted. Triggered by the observation of partially bunted ears with only a few bunt balls among otherwise healthy grains in field trials in Tulln, Austria, we adapted our scoring method by cutting ears at least two times, once in the upper third and once in the bottom third of the ear. Thereby, we achieved a more accurate scoring of incidence which covered partially bunted ears. In addition, we applied two more methods of bunt assessment, i.e. the number (BS) and weight (WBS) of bunt sori relative to the total number/weight of grains plus bunt sori in a sample. Combining these measures, we were able to determine in which genotypes partially bunted ears occurred more frequently than in others as the BS scores of such partially infected lines would be considerably lower than CB obtained from standard scoring. Our results confirm the observations already made by Sampson (1927) and Gieseke (1929) that partial bunt infections occur primarily in genotypes with a certain level of disease resistance. Both studies were conducted with a cultivar called ‘Heils Dickkopfweizen’ (syn. ‘Dornburger Heils Dickkopf’) which was known to harbor resistance to bunt. In our experiments, heavily infected genotypes had similar levels of BS compared to CB with BS scores sometimes even exceeding CB levels. These rare cases of BS > CB were due to the fact that scoring of CB and BS was not done as repeated measurements on the same ears. Instead, new ears on which BS and WBS were scored were randomly selected from non-cut ears in plots that had previously been assessed for CB. Genotypes showing at least some levels of resistance with CB scores below or around 30%, on the other hand, frequently had BS scores which were only up to 40% of the CB levels. Gassner (1938) attributed these observations to a kind of race between the fungus and the ovule taking place in early stages of grain development.

The lower weight of bunt sori compared to healthy grains was well reflected in WBS scores, which were in most cases 50-60% lower than BS scores. According to our results, assessing BS is sufficient to determine the extent of partial infections while additional scoring of WBS is not necessary as the two traits were highly correlated in both years of data collection. Based on the bunt characteristics assessed in our study, we were able to determine the extent of partially bunted ears but did not obtain data on partially bunted wheat grains. To draw further conclusions on partial infections of single grains, sowing healthy seeds harvested from partially bunted ears in field trials would be an appropriate strategy.

### 4.2 Variation between common bunt populations

Common bunt infections were observed in two test locations for this study: one in the north-east of Austria and one in the north-west of Czechia. The distance between these two locations is approximately 300 km, but both can be regarded as Central European environments located in the humid continental agro-climate zone of Europe. Two different, locally collected bunt populations which showed distinctly different virulence patterns against lines in the diversity panel were used for artificial inoculations in these two environments. High virulence against differential lines for *Bt1*, *Bt4* and *Bt6* was observed in the Czech Republic, while these genes were still effective against the Austrian bunt population (**Table 1**). The high infection levels on the *Bt5*-differential with the Czech inoculum are most likely the result of admixture in the seed sample as tests of the same inoculum on seed samples for the *Bt5*-differential line in Denmark did not yield any infection (Borgen et al., 2023b). Several cultivars tested in both locations were moderately to highly susceptible in Austria but resistant against the Czech inoculum. Although the source of resistance is not known for most of these cultivars, many of them might possess *Bt10* (confirmed for ‘Tillexus’ and ‘Tillstop’) or also *BtP* as the Austrian inoculum was virulent against these genes but they showed resistance in the Czech trials. A similar reaction was observed for ‘Tilliko’ which carries *BtZ* from a *Thinopyrum intermedium* translocation and which was recently proposed as differential line for this resistance gene (Borgen et al., 2023a).

Seventeen genotypes of the whole panel showed bunt resistance across locations. Among the multi-parent breeding lines selected with KASP-markers, most of those harboring the QTL on chromosome 1A were infected in Austria but resistant in the Czech Republic (**Figure 1**). Lines harboring combinations of QTL on chromosomes 1A and 1B or only the QTL on chromosome 1B, on the other hand, were more strongly affected by the Czech bunt population. Infection levels varied not only between locations but also between years: while CB incidence was approximately 50% elevated in Austria in 2022 compared to 2021, infection levels in the Czech Republic were lower in 2022. Some genotypes that had moderate levels of CB in the Czech trials in 2021 were resistant in the same location in 2022. These results indicate that breeding for common bunt resistance needs to be done with strong emphasis on regional adaptation. Although the two trial sites are not very far away from each other and have a similar climate, local bunt populations show clear differences in their virulence against various resistance sources (Borgen et al., 2023b).

### 4.3 Applicability and efficiency of marker-assisted selection

The molecular markers used to select bunt resistance QTL in multi-parent breeding lines in our diversity panel were not diagnostic for individual loci but rather flanking the region to which the QTL had been mapped. As far as possible, at least two markers, one on each end of the chromosomal region, were applied to select for a specific locus. Due to the complex pedigrees of the breeding lines with up to ten different genotypes per line, some markers which yielded good selection results in previous studies (Muellner et al., 2021; Lunzer et al., 2023b) were not informative in individual lines. The more parental genotypes are added to the pedigree, the higher the chance becomes that one of these genotypes has the same allele call for a certain marker as the original resistance donor. If this is the case, the polymorphism of this marker is lost and it cannot be used for MAS. In **Supplementary Table S1**, QTL for which MAS could be conducted only with a single marker are indicated. This was the case in 13 out of 27 multi-parent breeding lines. Selection accuracy is negatively affected if screening is performed with just one flanking marker per locus. Therefore, outliers and high variance in lines selected through MAS as shown in **Figure 3** were expected. Even if two markers per QTL are used for selection, recombination events could occur in the region between these markers, leading to a loss of resistance in individual positively selected lines. Despite these challenges, MAS was effective in our panel with negatively selected and unselected lines showing higher infection levels compared to positively selected ones (**Figures 1**, **3**). Combinations of two or more QTL in a single line led to, on average, higher resistance levels than inheritance of only one QTL, which is in line with the findings of Wang et al. (2019) and Muellner et al. (2020); Muellner et al. (2021).

### 4.4 Conclusions for organic breeding

As scores for BS were on average lower than for CB in our diversity panel, this indicates that partial infections of wheat ears were rather the rule than an exception. It should therefore be re-considered whether the qualitative scoring of bunt incidence done in most experiments at the moment is really the most appropriate method or if rather a scoring method, also taking partial infections into account, would provide better knowledge about resistance levels in different genotypes. A first step for improvement could be cutting wheat ears several times as it was done in this study to make sure that partial infections of single ears do not go undetected. This is more time-consuming than the standard scoring, but still less tedious than assessing BS or WBS.

Among the 46 cultivars and breeding lines from breeding companies that were tested for this study, only six cultivars and one breeding line showed resistance to common bunt across years and locations. Four out of them are U.S. cultivars selected for bunt resistance, i.e. ‘Blizzard’ (Sunderman et al., 1991), ‘Bonneville’ (Souza et al., 1995), ‘Deloris’ (Hole et al., 2004) and ‘UI SRG’ (Chen et al., 2012). Only two cultivars come from European breeding programs, one (i.e. ‘Aristaro’) indeed from an organic program. The other, i.e. ‘Unitar’, a breeding line developed at NARDI in Fundulea, Romania, carries a 1AL·1RS wheat-rye chromosome translocation, introduced via a cross between wheat and triticale. The line shows stable resistance against common bunt across a wide range of locations that is attributed to a gene on the rye chromosome (Ciuca et al., 2023). Resistance to bunt was also described for wild wheat species, wheat wild relatives (Mamluk, 1998; Babayants et al., 2006), and tritordeum (Rubiales et al., 1996). However, no *Bt* genes from these wild relatives were yet characterized or exploited widely in commercial breeding. This is in contrast to e.g. leaf rust resistance where ∼50% of the more than 80 described *Lr* genes were derived from alien species and some of them successfully exploited in commercial breeding programs (Kumar et al., 2022). When searching for resistance sources against bunt diseases, alien species and wheat wild relatives should not be neglected in pre-breeding programs and characterization of resistance genes. Resistance to common bunt is currently mainly a problem of organic wheat production due to the lack of effective organic seed treatments, however, the European Union is aiming to halve the use of pesticides and increase the share of organic farms by 2030 within its Green Deal. Some fungicides used today in conventional agriculture might therefore be banned in the future. Hence, the incorporation of resistance genes against bunt diseases shall be a general breeding target for sustainable wheat production.

The six multi-parent breeding lines identified as being resistant across years and locations in this study represent genotypes that could be directly used in commercial breeding programs. Breeding lines with such complex pedigrees have advantages because of their high variation in the elite genetic background, but these come with the drawback of high chances for losing polymorphic markers for MAS. The selection accuracy with a quarter of all selected lines being actually resistant (0-5% infection) in our diversity panel corresponds to other experiments for MAS in multi-parent breeding lines conducted at BOKU (unpublished data). As genotypes harboring multiple resistance loci have been shown to possess superior resistance to bunt infections in previous works (Wang et al., 2019; Muellner et al., 2020; Muellner et al., 2021) and also in this study, pyramiding of bunt resistance genes should be a major focus in organic breeding. To achieve such a stacking, informative molecular markers for the loci of interest are essential. Common bunt is a serious problem in organic wheat production. Based on the results of this study, breeding of resistant varieties should be conducted regionally and sped up through the application of MAS to secure further organic and sustainable wheat production.

## Data availability statement

The original contributions presented in the study are included in the article/**Supplementary Material**. Further inquiries can be directed to the corresponding author.

## Author contributions

HG: Conceptualization, Data curation, Funding acquisition, Methodology, Project administration, Resources, Writing – original draft, Writing – review & editing. ML: Investigation, Methodology, Visualization, Writing – original draft, Writing – review & editing. VD: Investigation, Methodology, Writing – review & editing. KP: Investigation, Methodology, Writing – review & editing. HB: Conceptualization, Supervision, Writing – review & editing.
